# Trafficking regulation of proteins in Alzheimer’s disease

**DOI:** 10.1186/1750-1326-9-6

**Published:** 2014-01-11

**Authors:** Shangtong Jiang, Yanfang Li, Xian Zhang, Guojun Bu, Huaxi Xu, Yun-wu Zhang

**Affiliations:** 1 Fujian Provincial Key Laboratory of Neurodegenerative Disease and Aging Research, School of Pharmaceutical Sciences, Xiamen University, Xiamen, Fujian 361102, China; 2 Institute of Neuroscience, College of Medicine, Xiamen University, Xiamen, Fujian 361102, China; 3 Department of Neuroscience, Mayo Clinic, Jacksonville, Florida 32224, USA; 4 Degenerative Disease Research Program, Sanford-Burnham Medical Research Institute, La Jolla, California 92037, USA

**Keywords:** α-secretase, Amyloid beta (A4) precursor protein, β-secretase, Beta-site APP-cleaving enzyme 1, γ-secretase, A Disintegrin and Metalloprotease 10, Alzheimer’s disease, Trafficking

## Abstract

The β-amyloid (Aβ) peptide has been postulated to be a key determinant in the pathogenesis of Alzheimer’s disease (AD). Aβ is produced through sequential cleavage of the β-amyloid precursor protein (APP) by β- and γ-secretases. APP and relevant secretases are transmembrane proteins and traffic through the secretory pathway in a highly regulated fashion. Perturbation of their intracellular trafficking may affect dynamic interactions among these proteins, thus altering Aβ generation and accelerating disease pathogenesis. Herein, we review recent progress elucidating the regulation of intracellular trafficking of these essential protein components in AD.

## Introduction

Alzheimer’s disease (AD) is a progressive neurodegenerative disorder characterized clinically by cognitive and memory dysfunction, accompanied by classical hallmark pathologies such as intraneuronal neurofibrillary tangles (NFTs) and extracellular amyloid plaques [1-3]. NFTs are enriched with hyperphosphorylated microtubule-associated protein tau (MAPT) [2], which can be phosphorylated by multiple protein kinases. Amyloid plaques comprise a heterogeneous population of proteolytically-generated small β-amyloid peptides (Aβ) [1,4,5]. Mounting evidence indicates that overproduction/aggregation of Aβ in the brain is a primary cause of AD. According to the “amyloid cascade hypothesis”, neurotoxic Aβ may trigger a cascade of complex neurodegenerative events leading to synaptic dysfunction, NFT formation and eventually neuron loss in affected brain areas [6-9].

Aβ is generated from amyloid beta (A4) precursor protein (APP). APP can be processed by amyloidogenic and non-amyloidogenic pathways. Amyloidogenic processing involves initial APP cleavage by β-secretase within the ectodomain, leading to the release of a soluble APP fragment called sAPPβ. The remaining membrane-anchored APP β-carboxyl-terminal fragment (β-CTF) is then cleaved by the γ-secretase complex within the lipid bilayer, resulting in the production of Aβ peptide and the APP intracellular domain (AICD). Alternatively, processing within the non-amyloidogenic pathway involves APP cleavage by α-secretase within the Aβ domain. The α-cleavage releases the extracellular domain of APP called sAPPα. The remaining membrane-bound fragment is called APP α-CTF and can be further processed by γ-secretase to generate AICD and a p3 peptide that is rapidly degraded [1,10]. Whether APP is processed through the amyloidogenic pathway by β-secretase or through the non-amyloidogenic pathway by α-secretase greatly depends on colocalization between APP and these secretases, which in turn is dependent on their subcellular co-distribution. Hence, comprehensive elucidation of the mechanisms responsible for regulating the intracellular trafficking of APP and related secretases are important aspects in our understanding of AD and AD pathogenesis.

### Regulated trafficking of APP

#### APP and its trafficking route within the cell

APP is a ubiquitously-expressed type I transmembrane protein belonging to a family comprising three members: APP, APP-like protein 1 and APP-like protein 2, all of which share a highly conserved extracellular region containing Kunitz protease inhibitor (KPI), E1 and E2 domains [11,12]. However, only APP contains the Aβ region. There are three major APP protein isoforms: APP770, APP751 and APP695. The longer isoforms APP770 and APP751 contain the KPI domain, whereas APP695 is devoid of this domain and is the predominant isoform expressed in the brain [13,14]. APP participates in multiple cellular events including cell adhesion, neurite outgrowth, neuronal migration, protein transport, cell signaling and synaptogenesis. For a more complete review on the function of APP please see [1].

**Figure 1:**
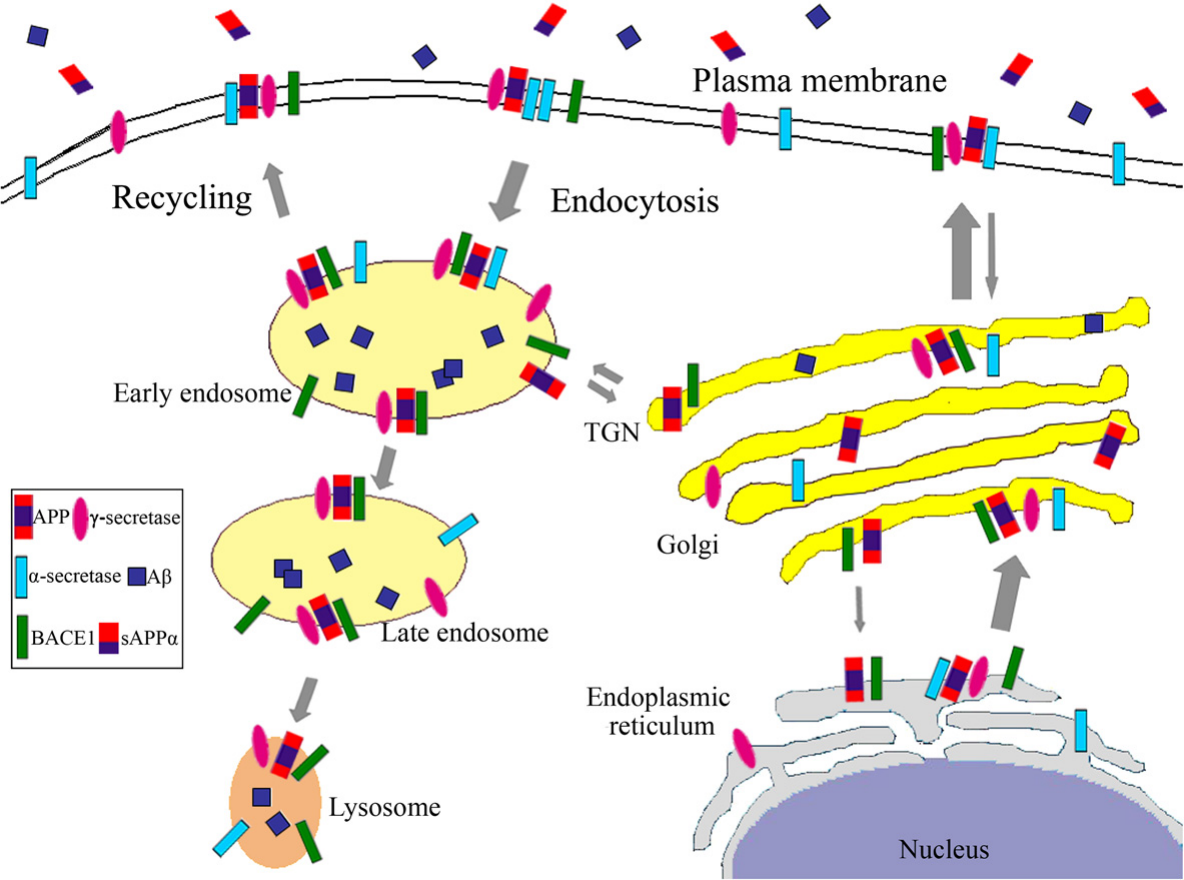
**Typical trafficking route of APP, BACE1, α-secretase and γ-secretase.** Newly synthesized APP, BACE1, α-secretase and γ-secretase traffic through the secretory pathway, from the ER to Golgi/TGN and to the plasma membrane, during which process retrieval may occur and APP may be cleaved to generate Aβ. At the plasma membrane APP is largely subjected to non-amyloidogenic processing by α-secretase to release sAPPα. Un-cleaved APP and various secretases at the plasma membrane may undergo endocytosis via early endosome to late endosome/lysosome for degradation. Acidic endosome/lysosome provides optimal environment for BACE1 activity and APP is mainly subjected to amyloidogenic processing for Aβ production in these compartments. In addition, a fraction of these proteins in endosome can be either recycled back to plasma membrane or retrieved back to TGN.

Full-length APP is synthesized in the endoplasmic reticulum (ER) and transported through the Golgi/trans-Golgi network (TGN) apparatus where APP undergoes posttranslational modifications such as glycosylation and phosphorylation during maturation. TGN is also the major site of resident APP in neurons [15-17]. Full-length APP can be transported to the cell surface in TGN-derived secretory vesicles. At the plasma membrane APP is either cleaved by α-secretase to produce sAPPα [18] or re-internalized within clathrin-coated vesicles to the endosome. APP in the endosome can either be recycled back to cell surface or be delivered to the lysosome for degradation (Figure 1) [19,20]. Aβ production involves multiple intracellular organelles [15-17,21,22]. Typically, increased APP delivery to or decreased APP internalization from the cell surface favors the non-amyloidogenic processing, whereas elevated retention of APP in acidic compartments such as endosomes promotes amyloidogenic processing and consequent Aβ production.

#### APP structure and post-translational modification affect its trafficking

The intracellular domain of APP is key in regulating APP trafficking through interactions with various cytosolic trafficking factors. A highly conserved YENPTY motif (amino acids 682–687 in APP695) within APP intracellular domain is the major binding site for a number of intracellular adaptors such as APP-binding, family A (APBA, previously called MINT) members and APP-binding, family B, member 1 (APBB1, previously called Fe65) [23]. Mutation within this motif can attenuate the internalization of APP, thereby decreasing Aβ generation [24]. Recently, the extracellular domain of APP has also been shown to affect its trafficking: the KPI domain located in the APP751 extracellular region promotes sorting of APP751 to the plasma membrane, accompanied by decreased Aβ formation in comparison to the neuronal enriched, KPI domain-absent APP695 isoform. Mutation in the KPI domain of APP751 results in its retention in the ER and elevated Aβ production [25].

APP can be phosphorylated at sites S655 and T668 (based on APP695 numbering) and APP phosphorylation may also affect its trafficking. A YTSI (amino acids 653–656 in APP695) motif within the APP intracellular domain is associated with APP internalization and Golgi polarized sorting in MDCK epithelial cells; and phosphorylation of S655 within this motif promotes APP retrieval from the endosome to TGN [26,27]. Phosphorylation of APP T668 facilitates amyloidogenic APP processing and Aβ generation [28]. Although direct evidence linking T668 phosphorylation and APP trafficking is scarce, T668 phosphorylation may interfere APP’s interaction with other proteins and thus potentially affect its processing/trafficking [29]. Indeed, phosphorylated APP is found to be preferentially enriched at nerve terminals and plasma membrane in neurons, and accumulates in pathological damaged regions of AD brains [30].

APP can be ubiquitinated for its proteasomal degradation. But ubiquitination of APP may also inhibit its endocytosis to endosomal compartment and thereby lower Aβ generation. In support of this notion, the E3 ubiquitin ligase component FBL2 can promote APP ubiquitination and suppress its endocytosis [31]. In addition, an ubiquitin-like protein, ubiquilin-1 (UBQLN1), can stimulate K63-linked polyubiquitination at APP K688 to inhibit APP maturation and delay APP proteolytic processing through APP sequestration primarily within the Golgi apparatus [32,33]. Interestingly, variants in the *UBQLN1* gene have been proposed to be associated with AD [34], implying that UBQLN1-mediated APP trafficking/processing might be altered in AD.

#### The LDLR family in APP trafficking

The low density lipoprotein receptor (LDLR) family consists of a number of cell surface receptors of diverse functions, including the LDLR, LDLR-related protein 1 (LRP1), LDLR-related protein 1B (LRP1B), LDLR-related protein 2 (LRP2), LDLR-related protein 4 (LRP4), LDLR-related protein 5 (LRP5), LDLR-related protein 6 (LRP6), LDLR-related protein 8 (LRP8, also known as APOER2), LDLR-related protein 10 (LRP10), LDLR class A domain containing 3 (LDLRAD3), sortilin-related receptor, (LDLR class) A repeats containing (SORL1, also known as SorLA or LR11), etc. [35,36]. Many LDLR family members act as receptors for apolipoprotein E (APOE), whose allelic polymorphisms are strongly associated with late-onset AD [37], further implicating the involvement of LDLR family members in AD. Recently several LDLR family members have been shown to modulate APP trafficking/processing. For example, LRP1 can bind APP directly or indirectly through the adaptor protein APBB1/Fe65 and promote rapid APP endocytosis to early endosomes to promote Aβ production [38,39]. In contrast LRP1B can compete with LRP1 for APP interactions and due to its comparatively slow rate of endocytosis compared to LRP1, LRP1B retains APP at cell surface and suppresses Aβ generation [40,41].

Another LDLR family member, LRP8/APOER2 can interact with APP intracellularly via APBA/MINT and DAB1 [42,43] or extracellularly via SPON1 (also known as f-spondin) [44]. APP trafficking and resultant non-amyloidogenic or amyloidogenic processing is dependent on the nature of LRP8/APOER2 and APP interactions (intracellularly or extracellularly) and specific linker proteins involved. A new member of LDLR family, LRP10, is found to shuttle between plasma membrane, TGN and endosome [45,46], and interacts with the APP ectodomain to sequester APP in Golgi compartments, thereby reducing APP β-processing and consequent Aβ generation [47].

The best characterized LDLR family member in AD is SORL1, a multifunctional neuronal receptor that binds APOE and APP. Genetic studies have identified multiple inherited variants in the *SORL1* gene associated with late-onset AD [48-51]. In addition, SORL1 level is decreased dramatically in frontal cortex and lymphoblasts from AD patients [52]. A correlation between decreased SORL1 expression and impaired cognitive function is also observed [53]. Although the exact physiological function of SORL1 has yet to be determined, its homology with sorting receptors that are involved in the transportation between plasma membrane, endosomes and Golgi suggests the protein trafficking function of SORL1 [54,55]. Indeed, SORL1 has been found to localize mainly in late endosomal and Golgi compartments and overexpression of SORL1 redistributes APP to the Golgi and reduces Aβ generation, whereas downregulation of SORL1 promotes APP sorting into Aβ-generating compartments to aggravate Aβ production [51,56-58]. These data indicate that SORL1 plays important roles in AD pathology by regulating the localization and processing of APP.

#### The RAB GTPase family in APP trafficking

RABs comprise a subfamily of small molecular weight GTPases within the RAS superfamily which regulates cellular trafficking. In humans, over 60 RAB family members have been described so far [59]. RABs function as molecular switches, alternating between active GTP-bound and inactive GDP-bound forms [60]. RABs regulate protein transport mainly by tethering/docking vesicles to target compartments [60]. Multiple RABs have been implicated in regulating APP trafficking. For example, RAB1B plays a key role in APP transport from the ER to Golgi. A dominant-negative RAB1B mutant blocks APP ER/Golgi transport and significantly inhibits Aβ secretion [61]. In addition, RAB6 functions as a negative regulator of APP anterograde transport through TGN or as a positive modulator of APP retrograde transport from post-Golgi vesicles back to TGN or Golgi cisternae; and a dominant-negative RAB6 mutant facilitates the anterograde transport of APP in the secretory pathway to promote α-cleavage [62]. Furthermore, RAB8 facilitates APP transport between the Golgi apparatus and plasma membrane and mutations in RAB8 results in the reduction of sAPPα secretion [63,64].

#### The SNX family in APP trafficking

Sorting nexins (SNXs) are a diverse group of cellular trafficking proteins that characteristically comprise a canonical phospholipid-binding domain. The ability of SNXs to bind specific phospholipids, as well as their propensity to form protein-protein complexes, suggests a role for these proteins in membrane trafficking and protein sorting [65,66]. Recently, several SNX members have been found to participate in APP metabolism/Aβ generation: SNX33 interacts with the endocytic GTPase dynamin and overexpression of SNX33 reduces the rate of APP endocytosis in a dynamin-dependent manner, leading to enhaced α-cleavage at the cell surface [67]. In addition, SNX17 can bind to APP in the early endosome and SNX17 downregulation causes a decline in steady-state levels of APP with a concomitant increase in Aβ production [68].

#### The APBA/MINT family in APP trafficking

The APBA/MINT family is an adaptor protein family comprising APBA1/MINT1, APBA2/MINT2 and APBA3/MINT3. All three proteins are enriched in neurons and contain a phosphotyrosine binding (PTB) domain in the central region and two tandem carboxyl-terminal PDZ (PSD-95, Drosophila disks-large, ZO-1) domains. Mounting evidence indicates that the APBA/MINT family is involved in multiple cellular activities relevant to neuronal protein transport and consequent synaptic function [69-71]. APBA/MINT proteins can interact with the APP YENPTY motif through their PTB domains to fine-tune APP trafficking and processing [70,71]. For instance, APBA1/MINT1 has been found to modulate both secretory and endocytic trafficking of APP and its metabolism [71]. In addition, APBA2/MINT2 can be phosphorylated by SRC to accelerate APP endocytosis and enhance APP sorting to autophagosomes, leading to enhanced intracellular Aβ accumulation. Conversely, an APBA2/MINT2 phospho-resistant mutant promotes APP trafficking in the recycling pathway to the cell surface, thus enhancing Aβ secretion [72]. Furthermore, APBA1/MINT1 or APBA2/MINT2 may function as a linker to mediate co-endocytosis of LRP8/APOER2 and APP, thereby elevating Aβ production [42,73]. Finally, APBA3/MINT3 also plays an obligatory role in mediating APP trafficking from the TGN to the plasma membrane and its deficiency reroutes APP trafficking to the endosomal/lysosomal pathway [74].

#### Other proteins involved in APP trafficking

In addition to general trafficking modulators, several other proteins have also been proposed to regulate APP trafficking. For example, we and others have shown that presenilin 1 (PS1), the catalytic component of the γ-secretase complex, can regulate APP intracellular trafficking: PS1 deficiency results in increased APP trafficking to the plasma membrane, whereas familiar AD-linked PS1 mutants dramatically reduce cell surface delivery of APP [75]. PS1 has been proposed to interact with APP and this might be a potential mechanism for its function in regulating APP trafficking. Alternatively, PS1 might regulate APP trafficking through interactions with trafficking factors such as RAB family members and phospholipase D1 (PLD1) [76-80]. PLD1 is a phospholipid-modifying enzyme regulating membrane trafficking events and PLD1 overexpression itself also promotes APP transport from TGN to the plasma membrane [79,80].

### Regulated trafficking of the β-secretase BACE1

#### BACE1 and its trafficking pathways within the cell

It is well accepted that beta-site APP-cleaving enzyme 1 (BACE1) is the dominant β-secetase enzyme for APP [81-84]. BACE1 is a 501 amino acid long type-I transmembrane aspartyl protease comprising a prodomain with significant homology to other aspartyl protease precursors. Overexpression or downregulation of BACE1 induces or inhibits APP processing and Aβ generation both *in vitro* and *in vivo*[81-85]. Several studies find that BACE1 level and activity are elevated significantly in AD brain [86,87]. In addition, in an APP overexpression AD mouse model, deletion of BACE1 can abolish Aβ pathology and rescue cholinergic dysfunction and memory deficits [88-90]. These findings suggest that BACE1 may be a good therapeutic target for AD treatment. On the other hand, more recent studies have found that in addition to APP, there are other BACE1 substrates including neuregulin 1 [91,92], p-selectin glycoprotein ligand-1 [93], ST6GAL1 [94], β-subunits of voltage-gated sodium channels [95], etc. This raises a possibility that inhibition of BACE1 might affect proper processing of these proteins and cause unwanted side effects. BACE1 null mice do develop several phenotypic abnormalities such as reduced body size, hyperactive behavior, decreased grip strength and elevated pain sensitivity [91,92,96]. Recently Eli Lilly’s BACE1 inhibitor LY2886721 was also found to cause liver toxicity during its Phase 2 clinical trial. However, it is unclear whether abnormal phenotypes found in BACE1 null mice are generated during developmental stages or during aging; and LY2886721-caused liver toxicity is not believed to be attributed to BACE1 inhibition. Furthermore, BACE1 heterozygous knockout mice have no reported abnormal phenotypes so far and heterozygous knockout of BACE1 still can reduce Aβ deposition in AD mice [97,98]. Hence, moderate inhibition of BACE1 activity may still be a good strategy for AD treatment.

Like other aspartic proteases, immature BACE1 containing the short prodomain is initially synthesized in the ER and subsequently transported to the Golgi apparatus where the prodomain is removed by FURIN or other FURIN-like proteases [99-102]. Removal of this prodomain leads to a ~2-fold increase of BACE1 activity [103]. BACE1 can also be N-glycosylated and mature BACE1 is exported either to the plasma membrane or to the endosomal compartment. Plasma membrane localized BACE1 can be internalized into the endosome, whereby BACE1 can be recycled to the plasma membrane or transferred to late endosomal/lysosomal compartments (Figure 1). As optimal BACE1 activity is achieved in acidified environments, BACE1 is mainly found in acidic subcellular compartments such as endosome [82,104-107]. Therefore, any modulator that can affect BACE1 shuttling between TGN, the plasma membrane and endosome may play a role in APP processing and Aβ generation.

#### The GGA family in BACE1 trafficking

Golgi-localized γ-adaptin ear-containing ADP ribosylation factor binding proteins (GGAs) are a family of monomeric clathrin adaptor proteins that participate in transport of cargo proteins from TGN to endosome [108]. Mammalian GGAs (GGA1, GGA2, and GGA3) all comprise an amino-terminal VHS (VPS-27, Hrs, and STAM), an intermediary GAT (GGA and Tom1) and a carboxyl-terminal GAE (γ-adaptin ear) domain [108]. VHS and GAT domains are highly conserved among GGA1, GGA2 and GGA3 [109]. Recruitment of GGAs from the cytosol to TGN is mediated by the GAT domain [109-111]. Subsequent GGA VHS domain binding to the acidic cluster-dileucine (ACDL) sorting motif within the cytoplasmic tails of selective cargo proteins then sorts the selected cargo into clathrin-coated vesicles that are delivered to the endosome [112].

The BACE1 DISLL (amino acids 496–500) sequence is an ACDL motif located in the BACE1 cytoplasmic domain and can be recognized by the GGA VHS domain [113,114]. Previous studies indicate that GGA1 modulates BACE1 retrieval from the endosome to TGN in a phosphorylation-dependent manner. Serine phosphorylation within the BACE1 DISLL motif enhances BACE1-GGA interaction [115]. Phosphorylated BACE1 is efficiently transported from endosomes to TGN, whereas non-phosphorylated BACE1 enters a direct recycling route to the plasma membrane [114,116,117]. Overexpression of GGA1 decreases Aβ secretion, while suppression of GGA1 conversely elevates Aβ secretion [116]. In addition, a recent study shows that GGA1 but not GGA2 or GGA3 can modulate SORL1 endocytic traffic and consequent APP traffic to the endocytic recycling compartment [118]. Furthermore, mutation of BACE1 S498 to a non-phosphorylatable residue enhances BACE1 targeting to SORL1-positive compartments [118]. In contrast, GGA3 is proposed to mediate targeting of BACE1 to lysosomes for degradation [119-121]. In AD brains, GGA3 level is significantly decreased and inversely correlated with elevated levels of BACE1. Downregulation of GGA3 also increases BACE1 levels [120,121]. Moreover, BACE1 can be ubiquitinated at K501 and a GGA3 mutant with reduced ubiquitin-binding ability fails to regulate BACE1 levels, implying that GGA3 may also regulate BACE1 via interactions with ubiquitin sorting machinery [122].

#### The retromer complex and the SNX family in BACE1 trafficking

The mammalian retromer complex is composed of VPS35, VPS29, VPS26, SNX1 and SNX2 [123]. Retromer is required for protein endosomal sorting and is closely linked to the endosome-to-Golgi retrieval pathway. Retromer was first identified to sort VPS10 from endosome back to the TGN in yeast [123]. Recent studies have identified some retromer interacting proteins including SORL1 [124] and BACE1 [125]. Suppression of VPS35 expression decreases BACE1 trans-Golgi localization and retains BACE1 in the endosome, the optimal environment for BACE1 activity. Consistently, heterozygous deletion of VPS35 in AD mice results in a significant increase in Aβ level in the mouse hippocampus, accompanied with AD-like phenotypes including cognitive memory deficits, defective long-term potentiation (LTP) and impaired postsynaptic glutamatergic neurotransmission in early adult age [125]. One recent study also found that downregulation of VPS35 in neurons results in increased colocalization of APP with the BACE1 in early endosomes and consequently elevated Aβ levels [126]. Similarly, downregulation of another retromer component VPS26 induces the accumulation of BACE1 in endosome [114]. Coincidently, the levels of VPS35 and VPS26 are found to be downregulated in AD [127].

The existence of two SNX family members, SNX1 and SNX2 within the retromer complex has provided a link for SNXs in regulating BACE1 trafficking. SNX6, a SNX2-like retromer regulator, can interact with BACE1 and negatively regulate its retrograde transport from early endosomes to TGN, retaining BACE1 in endosome [128]. We recently identified SNX12 as another BACE1-interacting protein and demonstrated its role in modulating BACE1 trafficking between the cell surface and early endosome [129].

#### Other proteins involved in BACE1 trafficking

There is evidence suggesting that endocytosis of APP and BACE1 are spatially separate: in contrast to clathrin-dependent APP endocytosis, BACE1 internalization to early endosome is found to be regulated by the small GTPase ADP ribosylation factor 6 (ARF6). Modulation of ARF6 levels or its activity affects endosomal sorting of BACE1 and altered APP processing/Aβ production. Furthermore, APP is cleaved by BACE1 in RAB5-positive early endosome and sorting of BACE1 from ARF6-positive towards RAB5-positive early endosome depends on the BACE1 DISLL motif [130].

In addition to its carboxyl-terminus, the transmembrane domain of BACE1 may also affect its trafficking. We recently found that the heavy components of the CUTA protein can interact with the BACE1 transmembrane region. Downregulation of CUTA decelerates BACE1 transport from Golgi/TGN to the plasma membrane and reduces steady-state levels of cell surface BACE1, thus resulting in increased Aβ generation [131]. Furthermore, BACE1 can interact with the reticulon family proteins and this is mediated by BACE1 transmembrane domain [132]. The reticulon family has four members, RTN1, RTN2, RTN3 and RTN4 (also known as Nogo), and is involved in various physiological processes such as membrane trafficking, axonal growth and apoptosis [133]. Overexpression of any reticulon protein has been shown to substantially reduce Aβ production. In neurons, BACE1 mainly colocalizes with RTN3, whose overexpression can block BACE1 in the ER and thus inhibit BACE1 activity for Aβ generation [134,135]. Interestingly, receptors of reticulon have also been found to mediate Aβ production but this is through their interaction with APP. Increased interaction between reticulon receptor and APP can reduce surface expression of APP and favors APP β-cleavage [136,137].

### Regulated trafficking of the γ-secretase complex

#### The γ-secretase complex

BACE1-digested APP CTFs are subsequently cleaved by the γ-secretase complex to release Aβ. In addition, γ-secretase cleaves a series of functionally important substrates such as NOTCH [138] and tyrosinase [139]. γ-secretase activity is produced from a high molecular weight complex consisting of at least four transmembrane components: presenilin (PS, with two mammalian homologs as PS1 and PS2), nicastrin, anterior pharynx-defective-1 (APH-1), and presenilin enhancer-2 (PEN2) [140,141]. PS1/2 have an undetermined number of transmembrane domains [142] and undergo endoproteolytic cleavage to generate PS amino-terminal fragments (NTFs) and CTFs. PS NTF and CTF form a functional heterodimer, each contributing a highly conserved aspartate residue indispensable for γ-secretase activity [143]. Nicastrin is a type I transmembrane glycoprotein considered to be the scaffolding protein within the γ-secretase complex. Nicastrin may also act as the γ-secretase receptor [144]. The seven-transmembrane APH-1 interacts with nicastrin to form a stable intermediate in an early assembly stage of the γ-secretase complex [140,141], whereas the two-pass transmembrane component PEN2 regulates PS endoproteolysis [145,146]. Each of these four components is necessary for γ-secretase activity and deficiency in any of these factors dramatically impairs the enzymatic activity.

#### Assembly of the γ-secretase complex and its trafficking route within the cell

Although aspects of γ-secretase complex formation and distribution have yet to be fully elucidated, a predicted model for the assembly, maturation, and trafficking of the γ-secretase complex has been proposed. In this model, the four components of the γ-secretase complex are synthesized in the ER, where they await incorporation into stable complex. Complex formation initially begins with the association of APH-1 and nicastrin to form an early intermediate subcomplex. Full-length PS is then incorporated into and stabilized within the complex. Subsequent assembly of PEN2 drives the conversion of the full-length PS into active NTF/CTF heterodimers in ER and Golgi compartments [140,147]. During its transit through Golgi/TGN, nicastrin undergoes complete glycosylation, thus transforming the γ-secretase complex into its mature form. The γ-secretase complex in large part shuttles between the ER and Golgi, whereas stabilized mature γ-secretase complex is transported to the plasma membrane. γ-secretase at the membrane can then undergo endocytosis to endosomes, and subsequent trafficking to late endosomes/multivesicular bodies or the lysosome (Figure 1) [148-151].

#### Factors regulating γ-secretase trafficking and activity

In addition to its function as the catalytic core of the γ-secretase complex, PS1 has been shown to modulate trafficking of several transmembrane proteins including APP [75]. Since PS1 interacts with a series of trafficking modulator proteins such as RAB family members and PLD1, PS1 might exert its trafficking regulation through these interactions [152]. Conversely, these trafficking modulators may also reciprocally regulate PS1 trafficking. For example, we find that PLD1 interacts with PS1 and overexpression of PLD1 promotes cell surface accumulation of PS1 in an APP-independent manner [153]. A trafficking regulation role has been proposed for APP [154] and we also find that APP can reciprocally regulate the intracellular trafficking of PS1 and other γ-secretase components. APP deficiency results in elevated transportation of PS1/γ-secretase from TGN to the cell surface, thereby enhancing NOTCH cleavage [153]. Additionally, APP can function as a kinesin-I membrane receptor and mediates axonal transport of PS1 and BACE1 [155].

The recently identified postsynaptic protein ARC also interacts with PS1 and participates in sorting PS1 to early and recycling endosomes to enhance amyloidogenic APP processing. Aβ production can be blocked by interrupting ARC association with PS1/γ-secretase and genetic deletion of ARC decreases Aβ load in a transgenic AD mouse model [156].

The G protein-coupled receptor (GPCR) β2-adrenergic receptor is associated with PS1 where agonist-induced β2-adrenergic receptor endocytosis mediates trafficking of PS1/γ-secretase to late endosomes and lysosomes to enhance Aβ production [157]. Another GPCR, GPR3 is also found to be a modulator of Aβ production: overexpression of GPR3 increases the formation and cell surface localization of the mature γ-secretase components, accompanied with stimulated Aβ generation but not NOTCH processing. In contrast, GPR3 deficiency prevents Aβ accumulation both *in vitro* and *in vivo*. Moreover, GPR3 is highly expressed in normal human brain regions implicated in AD and its level is elevated in sporadic AD brain [158]. Interestingly, recently it was suggested that both β2-adrenergic receptor and GPR3 exert their effects on Aβ generation through interacting with β-arrestin 2, a member of the arrestin family [159]. Arrestins are GPCR-interacting scaffold proteins and can inhibit G protein coupling to GPCRs, leading to GPCR desensitization and even mediating a G protein-independent signaling pathway [160]. β-arrestin 2 is found to interact with APH-1, redistribute the γ-secretase complex toward detergent-resistent membranes and increase its catalytic activity [159]. The degree of Aβ production stimulation is also correlated with GPCR-β-arrestin 2 binding and receptor trafficking to endocytic vesicles [161]. Moreover, a subset of GPCRs including GPR3 and PTGER2, are found to be associated with APP when internalized via β-arrestin 2, thereby increasing APP amyloidogenic processing as well [161]. Coincidently, another arrestin family member, β-arrestin 1, can interact with APH-1 and facilitate the formation of nicastrin/APH-1 subcomplex and mature γ-secretase complex. β-arrestin 1 expression is upregulated in the brains of sporadic AD patients and transgenic AD mice, whereas deletion of β-arrestin 1 in AD mice results in reduced Aβ generation, diminished Aβ pathology and improved cognition [162].

The retention in ER sorting receptor 1 (RER1), a membrane factor mediating protein retrieval from Golgi to the ER, has been found to regulate γ-secretase trafficking. In one study, RER1 was found to bind to the transmembrane region of nicastrin and retrieve unincorporated nicastrin from the cis-Golgi back to the ER. Since RER1 competes with APH-1 for nicastrin binding, depletion of RER1 resulted in elevated γ-secretase complex assembly/activity and increased Aβ secretion [163]. In another study, RER1 was found to bind to the first transmembrane domain of PEN2 and retrieve unassembled PEN2 to the ER [164]. Both studies suggest that RER1 may be critical in monitoring quality control during γ-secretase complex assembly. Recently, RER1 overexpression was also shown to decrease both γ-secretase localization on the cell surface and Aβ secretion, whereas RER1 downregulation had opposite effects [165]. Furthermore, increased RER1 levels could decrease mature APP and increase immature APP, resulting in less surface accumulation of APP [165].

ATP-binding cassette transporter-2 (ABCA2) is a component responsible for retrograde trafficking of lipoprotein-derived cholesterol from late endosome/lysosome to the ER, and has been genetically linked to AD [166,167]. It has been recently shown that ABCA2 depletion can reduce γ-secretase complex formation due to alterations in protein levels, post-translational modification, and subcellular localization of nicastrin, thus affecting the γ-processing of APP and Aβ generation [168].

### Regulated trafficking of the α-secretase ADAM10

#### α-secretase, ADAM10 and its trafficking route within the cell

α-secretase-mediated APP cleavage leads to the release of sAPPα, and competitively antagonizes APP amyloidogenic processing and Aβ generation. The α-secretase processing pathway is Protein Kinase C-dependent and its activity can be modulated by various molecules such as the purinergic P2Y2 receptor, pituitary adenylate cyclase activating polypeptide, muscarinic receptors and NMDA receptors [169-172]. In addition to APP, several other proteins including prion, NOTCH receptors, ephrins, tumor necrosis factor α and cadherins have also been identified as α-secretase substrates [119,173-175].

So far, several ADAM (A Disintegrin and Metalloprotease) family members including ADAM9, ADAM10 and ADAM17 have been implicated as potential APP α-secretases. These type-I transmembrane ADAM components are prominently expressed in adult central nervous system. However, the expression of ADMA10 is relatively high compare to those of ADAM9 and ADAM17 in neurons [176-178] and conditional knockout of ADAM10 in mouse neurons nearly abolishes neuronal sAPPα generation [179]. Together, this suggests that ADAM10 is the major functional α-secretase in neurons [180,181]. In AD patients, the protein level of ADAM10, α-secretase activity and consequent sAPPα levels are found to be decreased significantly in platelets and temporal cortex regions [182,183]. In contrast, ADAM9 and ADAM17 thus far have only been shown to participate in regulated α-secretase cleavage [181,184].

Newly-synthesized ADAM10 in the ER has a proprotein convertase recognition sequence between the prodomain and the catalytic regions. During its secretory trafficking to TGN and cell surface (Figure 1), ADAM10 can be cleaved by PC7 or FURIN to yield a mature, active form [185-187]. In neurons, α-secretase functions predominantly at the cell surface [188], with minor α-secretase activity observed in TGN [189-191].

#### Regulated trafficking of ADAM10

An arginine-rich (^723^RRR) sequence within the ADAM10 intracellular C-terminal tail is required for its retention in the ER and attenuates trafficking to the cell surface. Mutation of the second arginine within the ^723^RRR sequence is sufficient to allow ADAM10 exit from the ER and enhance ADAM10 distribution to the neuronal cell surface [192].

Protein interactions can influence ADAM10 localization. For instance, interactions with synapse-associated protein-97 (SAP97), a protein involved in dynamic transport of proteins to the excitatory synapses, facilitates ADAM10 targeting to the postsynaptic membrane. ADAM10-SAP97 interaction is triggered by NMDA receptor activation, thus promoting the non-amyloidogenic processing of APP. Conversely, disrupting ADAM10-SAP97 interaction redistributes ADAM10 localization from postsynaptic membranes, thereby suppresses non-amyloidogenic APP processing. As a consequence, this results in Aβ accumulation, MAPT hyperphosphorylation, and impaired behavioral performance and synaptic dysfunction in mice, thereby recapitulating early-stage AD phenotypes [193]. In the hippocampus of AD patients, the association of ADAM10 with SAP97 is found to be reduced [194], implying a dysfunction in SAP97-mediated ADAM10 sorting in AD. Interestingly, while long-term depression can promote SAP97-dependent ADAM10 distribution and activity in synaptic membranes, LTP can induce ADAM10 endocytosis and reduce ADAM10 activity through the association of the clathrin-adaptor AP2. Furthermore, ADAM10-AP2 association is found to be enhanced in AD patients [195]. These findings indicate that SAP97 and AP2 are critical components in controlling ADAM10 localization and activity at synapses to modulate pathophysiology in AD.

## Conclusions

Although APP is a well-characterized substrate of α-, β- and γ-secretases, the physiological relevance of the various APP processing pathways is far from clear. Amyloidogenic Aβ generation from APP clearly contributes to AD-associated neurotoxicity and plays a central role in the pathogenesis of AD. A wealth of experimental evidence demonstrates APP cleavage by different secretases at distinct cellular sites, implying the importance of the subcellular co-distribution of APP and various secretases in Aβ generation. Indeed, APP processing is a complicated and dynamic process during which a number of adaptors/linkers, motor molecules and membrane proteins work together to facilitate proper sorting of APP and its secretases. This review covers some of the salient aspects of intracellular trafficking regulation of APP and secretases. Future investigation is expected to enhance our understanding of how dysregulation of protein trafficking can contribute to AD pathogenesis and may potentially reveal new strategies in AD treatment.

## Abbreviations

Aβ: β-amyloid; ABCA2: ATP-binding cassette transporter-2; ACDL: Acidic cluster-dileucine; AD: Alzheimer’s disease; ADAM: A Disintegrin and Metalloprotease; AICD: APP intracellular domain; APBA: APP-binding, family A; APBB1: APP-binding, family B member 1; APOE: Apolipoprotein E; APP: Amyloid beta (A4) precursor protein; ARF6: ADP ribosylation factor 6; BACE1: Beta-site APP-cleaving enzyme 1; CTF: Carboxyl-terminal fragment; DS: Down syndrome; ER: Endoplasmic reticulum; GAE: γ-adaptin ear; GAT: GGA and Tom1; GGA: Golgi-localized γ-adaptin ear-containing ADP ribosylation factor binding protein; GPCR: G protein-coupled receptor; KPI: Kunitz protease inhibitor; LDLR: Low density lipoprotein receptor; LDLRAD3: LDLR class A domain containing 3; LRP1: LDLR-related protein 1; LRP1B: LDLR-related protein 1B; LRP2: LDLR-related protein 2; LRP4: LDLR-related protein 4; LRP5: LDLR-related protein 5; LRP6: LDLR-related protein 6; LRP8: LDLR-related protein 8; LRP10: LDLR-related protein 10; LTP: Long-term potentiation; NFTs: Neurofibrillary tangles; NTF: Amino-terminal fragment; PDZ: PSD-95, Drosophila disks-large, ZO-1; PLD1: Phospholipase D1; PS1: Presenilin 1; PTB: Phosphotyrosine binding; RER1: Retention in ER sorting receptor 1; SAP97: Synapse-associated protein-97; SNX: Sorting nexin; SORL1: Sortilin-related receptor, (LDLR class) A repeats containing; TGN: Trans-Golgi network; VHS VPS-27: Hrs and STAM; UBQLIN1: Ubiquilin-1.

## Competing interests

The authors declare that they have no competing interests.

## Authors’ contributions

SJ, YL, XZ, GB, HX and YWZ contributed to the writing and revising of the manuscript. All authors read and approved the final manuscript.
